# Peritoneal dialysis in an adult patient with tetralogy of Fallot diagnosed with incomplete Alagille syndrome

**DOI:** 10.1186/s12881-020-01134-7

**Published:** 2020-10-02

**Authors:** Malgorzata Ponikowska, Agnieszka Pollak, Ewa Kotwica-Strzalek, Dorota Brodowska-Kania, Magdalena Mosakowska, Rafal Ploski, Stanislaw Niemczyk

**Affiliations:** 1 Department of Internal Diseases, Nephrology and Dialysis, Military Institute of Medicine, 128 Szaserów St, 04-141 Warsaw, Poland; 2 Department of Molecular Biotechnology, Faculty of Chemistry, University of Gdańsk, Wita Stwosza 63 St., 80-308 Gdansk, Poland; 3 Department of Medical Genetics, Medical University of Warsaw, 3c Pawinskiego St., 02-106 Warsaw, Poland

**Keywords:** Alagille syndrome, *JAG1* mutation, Tetralogy of Fallot, End-stage renal disease, Peritoneal dialysis

## Abstract

**Background:**

Alagille syndrome is an autosomal dominant disorder usually caused by pathogenic variants of the *JAG1* gene. In the past, cholestasis was a condition sine qua non for diagnosis of the syndrome. However, recent advancements in genetic testing have revealed that clinical presentations vary from lack of symptoms, to multiorgan involvement. Tetralogy of Fallot, the most frequent complex congenital heart defect in Alagille Syndrome, very rarely leads to renal failure requiring dialysis – there are only single reports of such cases in the literature, with none of them in Alagille Syndrome.

**Case presentation:**

A 41-year-old woman suffering from cyanosis, dyspnea and plethora was admitted to the hospital. The patient suffered from chronic kidney disease and tetralogy of Fallot and had been treated palliatively with Blalock-Taussig shunts in the past; at admission, only minimal flow through the left shunt was preserved. These symptoms, together with impaired mental status and dysmorphic facial features, led to extensive clinical and genetic testing including whole exome sequencing. A previously unknown missense variant c.587G > A within the *JAG1* gene was identified. As there were no signs of cholestasis, and subclinical liver involvement was only suggested by elevated alkaline phosphatase levels, the patient was diagnosed with incomplete Alagille Syndrome. End-stage renal disease required introduction of renal replacement therapy. Continuous ambulatory peritoneal dialysis was chosen and the patient’s quality of life significantly increased. However, after refusal of further treatment, the patient died at the age of 45.

**Conclusions:**

Tetralogy of Fallot should always urge clinicians to evaluate for Alagille Syndrome and offer patients early nephrological care. Although tetralogy of Fallot rarely leads to end-stage renal disease requiring dialysis, if treated palliatively and combined with renal dysplasia (typical of Alagille Syndrome), it can result in severe renal failure as in the presented case. There is no standard treatment for such cases, but based on our experience, peritoneal dialysis is worth consideration. Finally, clinical criteria for the diagnosis of Alagille Syndrome require revision. Previously, diagnosis was based on cholestasis – however, cardiovascular anomalies are found to be more prevalent. Furthermore, the criteria do not include renal impairment, which is also common.

## Background

Alagille syndrome (ALGS) is an autosomal dominant, multiorgan disorder caused by aberrations in the Notch-signaling pathway. Its prevalence is estimated to be between 1 in 70, 000 and 1 in 30,000 [1]. Ninety-seven percent of cases are caused by pathogenic variants of the *JAG1* gene (20p12) with < 1% of cases resulting from mutations in the *NOTCH2* gene (1p13) [2].

When the disease was first described in 1969, neonatal liver disease with conjugated hyperbilirubinemia was recognized as a predominant feature. According to classic criteria, a definitive diagnosis required the presence of interlobular bile duct paucity together with at least three out of five of the following clinical features: chronic cholestasis, cardiac disease, ocular abnormalities, dysmorphic face and skeletal abnormalities. Subsequently, additional symptoms typical for ALGS were identified – they included: renal and vascular anomalies, growth retardation and pancreatic insufficiency [2]. However, they have never been formally included in the diagnostic criteria. As genetic testing has advanced, it has been shown that pathogenic variants of the *JAG1* and *NOTCH2* genes may have a wide variety of clinical presentations ranging from lack of symptoms to multiorgan involvement [3].

Tetralogy of Fallot (ToF) is the most frequent complex congenital heart defect in ALGS, present in 11% of cases [4, 5]. ToF is caused by anterosuperior displacement of the conal septum which results in an overriding aorta, ventricular septal defect and right ventricular hypertrophy secondary to pulmonary stenosis. ToF may be isolated, however, in 20% of cases, it is present as a part of genetic syndromes such as DiGeorge, Down, Opitz, Noonan, etc. [6] A small number of ToF cases (1.3–2.1%) are associated with pathogenic variants of *JAG1* [7, 8]. Ongoing research links the remaining cases to variants in genes including *NKX2.5*, *GATA4*, *FOXC2*, *TBX5* and *TBX1* among others [6].

End-stage renal disease (ESRD) requiring dialysis is an extremely rare complication in ToF – there are only individual reports of such cases in the literature, although none of them in ALGS. There is no standard treatment for such cases.

## Case presentation

A 41-year-old woman presented to our department with cyanosis, dyspnea and plethora. The patient suffered from chronic kidney disease (CKD) and ToF, for which she was treated palliatively in the past. On admission, her oxygen saturations were 66.6% on room air. Blood tests revealed a red blood cell count of 7.54 × 10^12^/L, hemoglobin 21.8 g/dL and hematocrit as high as 71%. Ferritin level was 73 ng/mL. Liver function tests revealed bilirubin levels to be 0.9 mg/dL, aspartate aminotransferase 50 U/L and alanine aminotransferase 43 U/L. A creatinine level of 3.5 mg/dL and urea 171 mL/dL indicated ESRD. A total calcium amount of 8.2 mg/dL, inorganic phosphates 6.5 mg/dL and parathyroid hormone level of 748.7 pg/mL were suggestive of secondary hyperparathyroidism. Metabolic acidosis was recognized with a pH of 7.196 and bicarbonates at 13.6 mmol/L. An ultrasound scan showed horseshoe kidney and liver with increased echogenicity.

**Fig. 1:**
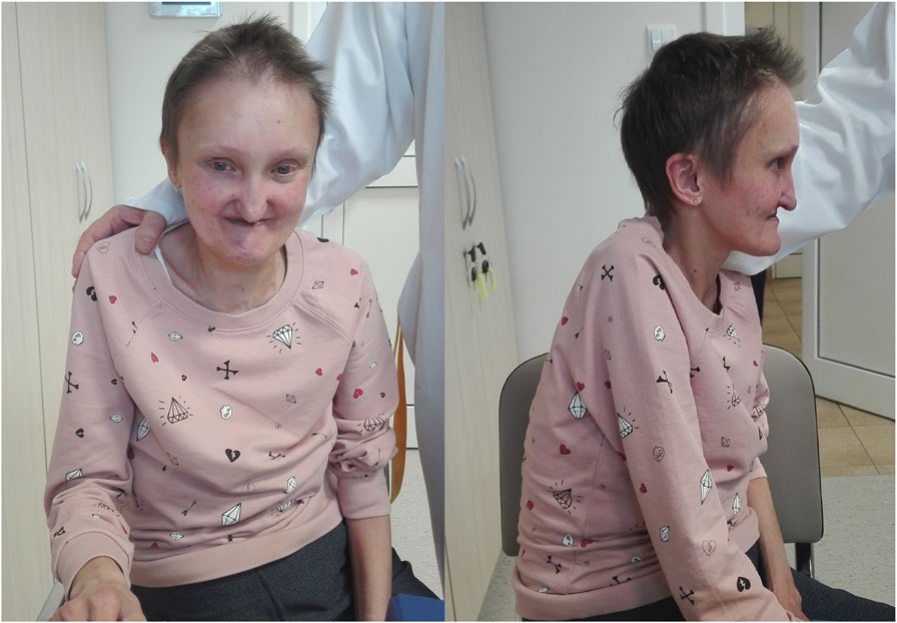
Dysmorphic facial features of the patient: prominent forehead, deep set eyes, pointed and small chin, hypertelorism

Dysmorphic facial features were also observed, which included a prominent forehead, deep set eyes, pointed and small chin and hypertelorism (Fig. 1). These characteristics, combined with horseshoe kidney, were highly suggestive of an undefined genetic syndrome, prompting further clinical investigation and genetic testing. Imaging revealed thoracic scoliosis and a deformed thoracic cage with decreased anterioposterior diameter. Skeletal anomalies typical of ALGS, such as butterfly vertebrae, were not observed. Ophthalmologic examination showed grade 3 hypertensive retinopathy and dry eye syndrome. It is worth mentioning that the patient had no family history of any genetic disease.

The patient’s physical development was impaired and, as a consequence, intellectual abilities were also diminished. A complex medical history resulting in social isolation, together with low socioeconomic status, likely impeded the patient’s learning and development potential.

Before admission to our department, the patient had already had an extensive past medical history. Some of her first symptoms, including cyanosis, were present at birth, however, neonatal jaundice was not reported. The diagnosis of ToF was made at the age of 12. Due to severe hypoplasia of the pulmonary vascular bed, a Blalock-Taussig shunt (a shunt between the right subclavian artery and right pulmonary artery) was performed on the right side. After a short period of observation, the shunt became occluded, and thus at the age of 14, a Blalock-Taussig shunt was applied on the left. When the patient was 33, she underwent multiple venesections due to grossly elevated hemoglobin levels (25.0 g/dL) and a hematocrit of 73.5%. However, phlebotomies had little effect on these parameters and were soon discontinued. Five years later, due to narrowing of the left shunt to 3 mm, stent implantation was performed. The intervention resulted in shunt flow with a maximal gradient of 124 mmHg. SaO_2_ increased from 68 to 82%. In contrast, the decreases in hemoglobin and hematocrit were deemed insignificant. Computed tomography (CT) of the heart performed at that time revealed ventricular septal defect of 10 mm in the membranous part of the septum, an overriding aorta with tricuspid aortal valve, very pronounced hyperplasia of the right ventricle, pulmonary stenosis, hypoplasia of the pulmonary trunk and extrapulmonary parts of the pulmonary arteries and an enlarged right atrium. When the patient was 41 years old, restenosis of the left shunt began to occur, likely due to pronounced polycythemia. In the following years, balloon angioplasty was performed twice. However, neither shunt flow nor the patient’s clinical condition improved significantly. The hemoglobin and hematocrit levels did not decrease and remained stable at 22 g/dL and 70%, respectively. Minimal flow through the left shunt was preserved. Lastly, echocardiography that the patient had at the age of 44 confirmed hyperplasia of the right ventricle (thickness of 12 mm) with preserved, good contractility (TAPSE of 23 mm). Very slight tricuspid regurgitation was visible together with signs of pulmonary hypertension (S’tr 8 cm/s).

Due to central cyanosis, the patient was suffering from repeated episodes of loss of consciousness and vertigo. She also had hypothyroidism. Moreover, the patient had suffered from hypertension since her late twenties. At the beginning, a combination of an ACE inhibitor, beta blocker and diuretic was used, but shortly after the initiation of treatment, the ACE inhibitor and diuretic were discontinued due to hyperkalemia. Her medication was limited to the beta blocker. This was sufficient until the age of 36, when the patient’s renal function started deteriorating. The patient and her mother claimed that values of blood pressure at home would fall below 140/90, but measurements at our department varied between 130/65 and 180/73 mmHg. They were performed on the patient’s leg due to the patient’s slim arms. At that time, treatment was limited to furosemide, bisoprolol and clonidine. Hepatitis and HIV screens were negative, although the patient had a record of HCV infection in the past.

With respect to the liver, bilirubin levels always remained within normal range during the 4 years of treatment at our department. Aspartate and alanine aminotransferase, though slightly elevated on admission, also remained within normal range most of the time. However, on several occasions, alkaline phosphatase was elevated, reaching values as high as 754 U/L during episodes of peritonitis.

Over time, progression to ESRD was observed. Proteinuria was not very pronounced with levels ranging between 100 and 500 mg/dL, while residual diuresis was 500 mL. Renal biopsy was never performed because the patient did not consent to it. When the patient was admitted to our department at the age of 41, renal replacement therapy was introduced. Continuous ambulatory peritoneal dialysis (CAPD) was chosen. After the introduction of CAPD, diuresis decreased to 200 mL. The mother was taught to successfully assist with CAPD. Acidosis was not compensated despite CAPD. The patient had regular appointments at the dialysis station. At the age of 44, two episodes of peritoneal dialysis-related peritonitis occurred, with the second episode being complicated by an ischemic stroke of the left hemisphere. Although it was successfully managed with conservative treatment, in the next 2 months, the overall condition of the patient gradually deteriorated. Both the patient and her family refused further hospitalizations. She died at the age of 45 and an autopsy was not performed.

## Methods

DNA from a proband and her family was extracted from peripheral blood using a standard protocol. Library preparation for whole exome sequencing (WES) was performed on the proband’s DNA sample with SeqCap EZ MedExome probes (Roche, Basel, Switzerland) targeting the human exome with enhanced coverage for clinically relevant genes. Subsequently, paired-end index sequencing (2 × 100) was performed on the HiSeq 1500 sequencer (Illumina Inc., San Diego, CA, USA) to obtain 99,763,149 reads (93% of target bases were covered at a minimum of 20x, whereas 98% had coverage at a minimum of 10x). The obtained raw data was processed using the pipeline described previously [9]. Briefly, after the bioinformatic analysis, all variants were filtered for coding, non-synonymous variants with population frequencies below 1% in the gnomAD database, as well as our in-house database (> 3500 exomes). Subsequently, all potentially relevant variants were evaluated regarding their possible pathogenicity with in silico predictions. Furthermore, the data was filtered for the occurrence of known, pathogenic variants described in the ClinVar database (https://www.ncbi.nlm.nih.gov/clinvar/). Variants were inspected with the Integrative Genomics Viewer (IGV). We did not discover any other variants in the exome data, apart from *JAG1* c.587G > A, that were potentially relevant and consistent with symptoms. Since we performed exome sequencing only for the proband, we did not establish the status of variants other than *JAG1* c.587G > A (de novo or inherited). The presence of the identified variant was verified by deep amplicon sequencing (DAS) in the proband and her relatives.

## Results

**Fig. 2:**
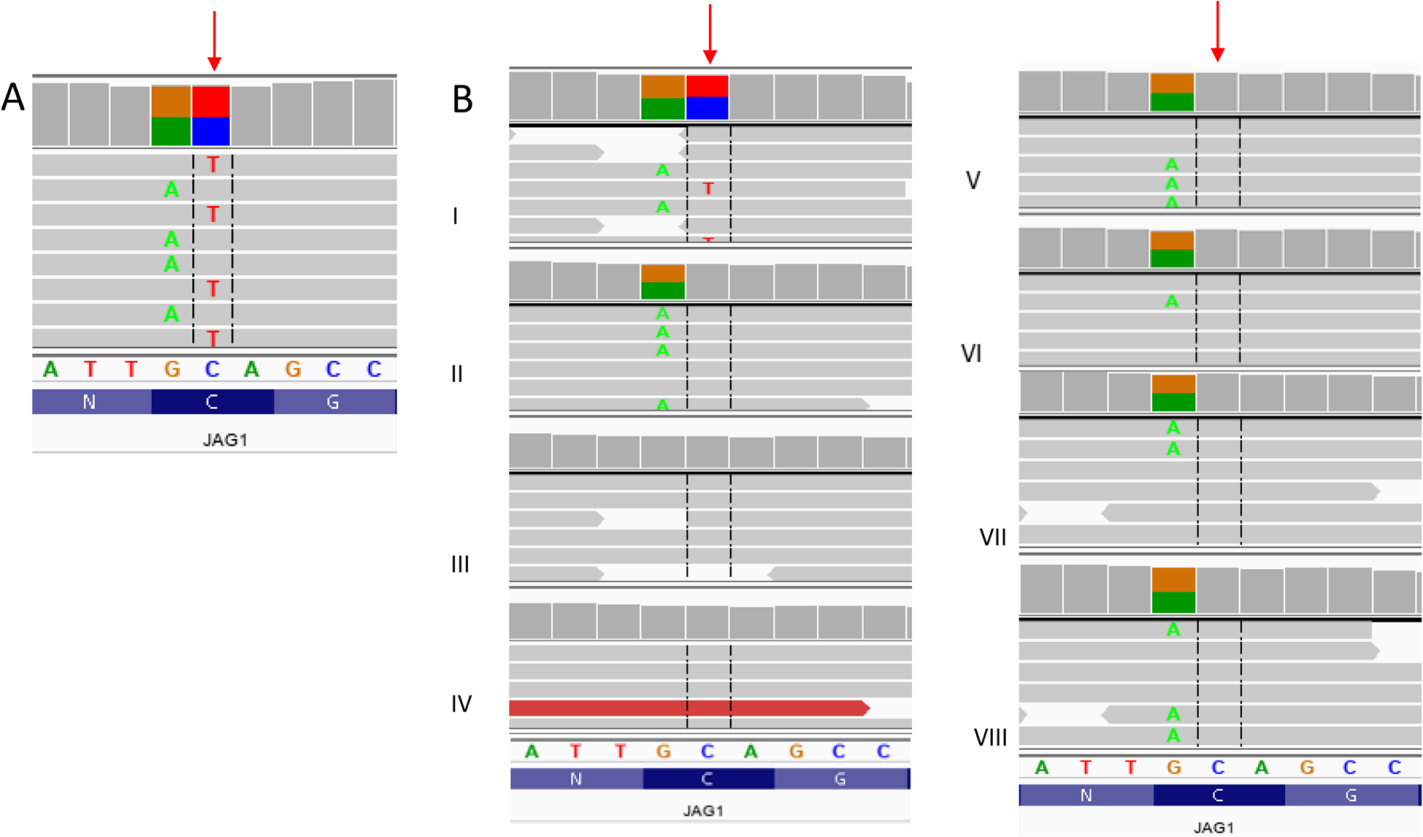
**a** Results of WES in the proband, **b** results of deep amplicon sequencing in the proband and her relatives (I – proband, II – mother, III – father, IV-VIII – siblings) Red arrow indicates c.587 position in the *JAG1* gene

The heterozygous missense mutation in the *JAG1* (NM_000214.2:c.587G > A) gene was prioritized for further validation. The population frequency of the variant was 0 in all tested databases, including gnomAD (v.2.0.2, https://gnomad.broadinstitute.org/) and an in-house database of > 1000 WES data from Polish individuals. DAS confirmed the presence of the heterozygous c.587G > A *JAG1* variant in the proband’s DNA and its absence in all of the tested family members of the proband (i.e. both parents and five siblings, Fig. 2). Thus, the family analysis demonstrated the de novo status of the c.587G > A variant. In silico analysis of pathogenicity using the CADD, MutationTaster, FATHMM, MetaSVM, MetalR, SIFT and Provean algorithms indicated that the c.587G > A variant is a disease-causing mutation.

The detected *JAG1* variant has not been described thus far (The Human Gene Mutation Database v. 2019.1, www.hgmd.cf.ac.uk), although other deleterious mutations within the *JAG1* gene are known to cause ALGS 1 (MIM#118450) with an autosomal dominant mode of inheritance. The novel JAG1 variant has been submitted to the Leiden Open Variation Database (ID: 00310041, https://databases.lovd.nl/shared/individuals/00310041).

## Discussion and conclusions

In the presented case, we identified a previously unknown missense variant – c.587G > A – within exon 4 of the *JAG1* gene, which results in an amino acid alteration (p.Cys196Tyr) in the DSL domain of JAG1 that is responsible for its binding to the Notch2 receptor [10]. Exon 4 is the second most frequent location of pathogenic variants within the *JAG1* gene [11].

Cysteine loss within the JAG1 protein frequently results in missense mutations in ALGS patients [10], likely due to the unique type of bonds (disulfide bonds) that cysteine forms. Disulfide bridges between cysteine molecules are crucial for the stability of the tertiary and quaternary structures of proteins and hence, for their function. The structure and function of a protein is very likely to be perturbed if a cysteine residue is substituted with tyrosine, as the latter is an aromatic amino acid with hydrophobic properties. A missense mutation c.686G > A causing the p.Cys229Tyr amino acid alteration, and thus disruption of the binding properties of the DSL domain, was reported in a patient with ALGS [12]. In vitro testing showed that this variant causes perinuclear retention of the modified protein and the inability to activate Notch signaling [10]. We hypothesize that the variant discovered in this patient case, which alters the DSL domain in a similar manner, is causative for the presenting phenotype.

In addition, two pathogenic variants were discovered in direct proximity to the one presented (at positions 588 and 582) in clinically diagnosed ALGS patients [13]. They resulted in premature termination codons, whereas the variant discovered in our patient, who had incomplete ALGS, leads to substitution of cysteine for tyrosine. This corresponds to claims of some authors that subclinical presentations of ALGS are more frequently caused by missense mutations, rather than deletions and other truncating variants that are typical of complete ALGS [7].

The expressivity of *JAG1* variants is variable and thus correlation between genotype and phenotype is dubious [2, 14]. In a study where 53 variant-positive relatives of ALGS patients were assessed, it was shown that 25 of them (47%) did not meet the classical clinical criteria, and moreover, two patients (4%) were completely symptom-free [3]. In the absence of obvious liver disease, which is the hallmark of ALGS, it took years to correctly diagnose our patient as her main symptom was ToF. However, the liver disease could have been subclinical. Aspartate and alanine transferase oscillated within the high – normal range, but we noted elevated alkaline phosphatase levels, especially during episodes of peritonitis, and a slight increase of liver echogenicity in the ultrasound. Knowing there have been cases of bile duct paucity with no abnormalities revealed in laboratory and imaging examination [15], we cannot exclude that our patient had silent bile duct paucity. Previously, cholestasis was condition sine qua non for the diagnosis of ALGS and now it is known to be present in only 89% of cases [1], whereas cardiovascular anomalies are found in up to 94% of patients [4]. Some researchers suggest that the developing heart might be more prone to decreased JAG1 dosages compared to liver [16]. Clinicians should therefore be urged to treat cardiovascular abnormalities as equally important as liver involvement when diagnosing ALGS.

Although surgical repairs for treating ToF are considered “curative”, long-term follow-ups show that despite the operations, patients experience complications caused by hemodynamic changes [17, 18]. The prevalence of significant renal impairment (GFR < 60) is 35-fold higher in cyanotic patients with congenital heart defects, even if they are treated, than in a general population [19]. This phenomena of co-occurrence of renal impairment secondary to chronic heart disease is known as cardiorenal syndrome, type 2 [20]. Bearing in mind the mild proteinuria in our patient, cardiorenal syndrome could have been one of the possible mechanisms contributing to the deterioration of renal function.

Other mechanisms in which cyanotic heart defects damage kidneys include poor blood oxygenation followed by polycythemia and increased blood viscosity. In patients with congenital heart disease, cyanosis doubles the risk of significant renal impairment [19]. Twenty years of follow-up of patients with ToF treated with a Blalock-Taussig shunt showed that hematocrit levels correlate with increasing filtration fraction [21], which in turn, leads to structural changes in the glomeruli, such as glomerulosclerosis [22, 23]. Our patient suffered from cyanosis throughout the major part of her life and at the age of 33, she had hematocrit as high as 73.5% – based on the aforementioned data, her filtration fraction could have exceeded 40% [21]. Although none of the patients treated with a Blalock-Taussig shunt developed significant renal impairment in the aforementioned study [21], and another study denies increased prevalence of CKD in patients with polycythemia [24], the observation periods in both cases could have been too short. Therefore, it cannot be excluded that long lasting polycythemia may be associated with severe deterioration of renal function. We suspect polycythemia played an important role in the development of ESRD in our patient.

Polycythemia is also associated with increased prevalence of hypertension [24] and we suspect it could have been the reason for the early onset of hypertension in our patient. Although hypertension is a known cause of CKD, blood pressure in our patient was well-controlled until renal function started to deteriorate and even later, it never reached values which could lead to ESRD if not accompanied by other factors. We believe that pre-existing renal anomalies due to ALGS were more important in the development of ESRD in our patient.

A recent retrospective cohort study involving pediatric patients with pathogenic variants of *JAG1* showed renal involvement in 39% of cases. The most common findings included renal dysplasia (23% of patients) and renal tubular acidosis (9.5% of patients) [25]. Our patient suffered from horseshoe kidney with increased echogenicity (accounting for renal dysplasia) and acidosis refractory to dialysis – likely due to renal tubular acidosis. Knowing that isolated ToF very rarely leads to ESRD [26], we suspect that renal abnormalities must have been an important precondition for it. According to the pediatric study, deterioration of renal function starts early – in underage patients with any form of renal involvement, CKD is present in 5.4%, and ESRD in 4.1% of cases [25]. Though common and typical in patients with pathogenic variants of *JAG1*, renal abnormalities are not included in the classic criteria for ALGS. What is more, variants of *JAG1* are present in 43, 51, 47 and 86% of patients corresponding to 2, 3, 4 and 5 systems affected [27]. Knowing these numbers, the need for full revision of the clinical diagnostic criteria for ALGS becomes clear.

Since only several case reports on ESRD requiring dialysis in ToF have been published, and none in ALGS, we faced a major challenge in choosing the appropriate therapeutic strategy. Renal or combined kidney-heart-lungs transplant were impossible due to significant hemodynamic and vascular stress. In addition, the intellectual abilities of the patient posed a contraindication as full cooperation on part of the patient was not guaranteed. Hemodialysis would have required the creation of a vascular access, which is a hemodynamically stressful event and could result in cardiac failure. Central line was not considered due to risk of infections and difficulties with maintaining patency in view of extreme polycythemia. Furthermore, polyglobulia may disable adequate heparinization which could lead to both clotting of the blood within the hemodialysis machine and embolism in the patient. What is more, the distance between the dialysis unit and the patient’s place of residence could be a major obstacle in achieving effective treatment. Given these factors, the final viable therapeutic tool was CAPD, which reduces cardiovascular stress because there is minimal variation in circulating blood volume. We were concerned about the intellectual abilities of the patient, but good cooperation with the mother allowed for successful peritoneal care at home for over 4 years and significantly enhanced the patient’s quality of life.

Although ToF rarely leads to ESRD requiring dialysis, if treated palliatively and combined with renal dysplasia (typical for ALGS), it can result in severe renal failure as in the presented case. Therefore, clinicians should aim at early recognition of *JAG1* pathogenic variants – firstly, to offer genetic counseling to patients and secondly, to predict the course of the disease. We suggest that pathogenic variants of *JAG1* should always be considered in ToF patients and that they should be offered early nephrological care. We also suggest considering use of CAPD in the treatment of ALGS patients who have ESRD and severe cardiac involvement such as ToF.

Finally, clinical criteria for the diagnosis of ALGS require revision in view of cardiovascular anomalies being more prevalent than liver involvement and common renal impairment in the condition.

## Data Availability

WES data was submitted to BioProject database (BioProject ID PRJNA665221, http://www.ncbi.nlm.nih.gov/bioproject/665221). JAG1 variant has been submitted to the Leiden Open Variation Database (ID:00310041, https://databases.lovd.nl/shared/individuals/00310041). Databases analyzed in the study included gnomAD (v.2.0.2, https://gnomad.broadinstitute.org/), ClinVar database (https://www.ncbi.nlm.nih.gov/clinvar/) and The Human Gene Mutation Database (v. 2019.1 https://www.hgmd.cf.ac.uk).

## Abbreviations

ALGS: Alagille syndrome
ToF: Tetralogy of Fallot
ESRD: End-stage renal disease
CKD: Chronic kidney disease
CAPD: Continuous ambulatory peritoneal dialysis
WES: Whole exome sequencing
IGV: Integrative Genomics Viewer
DAS: Deep amplicon sequencing
